# Germinal Matrix-Intraventricular Hemorrhage: A Tale of Preterm Infants

**DOI:** 10.1155/2021/6622598

**Published:** 2021-03-16

**Authors:** Walufu Ivan Egesa, Simon Odoch, Richard Justin Odong, Gloria Nakalema, Daniel Asiimwe, Eddymond Ekuk, Sabinah Twesigemukama, Munanura Turyasiima, Rachel Kwambele Lokengama, William Mugowa Waibi, Said Abdirashid, Dickson Kajoba, Patrick Kumbowi Kumbakulu

**Affiliations:** ^1^Department of Paediatrics and Child Health, Faculty of Clinical Medicine and Dentistry, Kampala International University, Uganda; ^2^Department of Surgery, Faculty of Clinical Medicine and Dentistry, Kampala International University, Uganda; ^3^Department of Surgery, Faculty of Medicine, Mbarara University of Science and Technology, Uganda

## Abstract

Germinal matrix-intraventricular hemorrhage (GM-IVH) is a common intracranial complication in preterm infants, especially those born before 32 weeks of gestation and very-low-birth-weight infants. Hemorrhage originates in the fragile capillary network of the subependymal germinal matrix of the developing brain and may disrupt the ependymal lining and progress into the lateral cerebral ventricle. GM-IVH is associated with increased mortality and abnormal neurodevelopmental outcomes such as posthemorrhagic hydrocephalus, cerebral palsy, epilepsy, severe cognitive impairment, and visual and hearing impairment. Most affected neonates are asymptomatic, and thus, diagnosis is usually made using real-time transfontanellar ultrasound. The present review provides a synopsis of the pathogenesis, grading, incidence, risk factors, and diagnosis of GM-IVH in preterm neonates. We explore brief literature related to outcomes, management interventions, and pharmacological and nonpharmacological prevention strategies for GM-IVH and posthemorrhagic hydrocephalus.

## 1. Introduction

Germinal matrix-intraventricular hemorrhage (GM-IVH) remains a devastating neurological complication with considerable mortality [1] and neurodevelopmental disability [2]. Hemorrhage originates in the capillary network of the subependymal germinal matrix (GM) of the developing brain and may disrupt the ependymal lining and progress into the lateral cerebral ventricle [3, 4]. Although significant strides in obstetrics and neonatal medicine have led to improved survival of preterm infants with lower gestational age and birth weight [5–7], we seem to have reached the nib of our ability to ensure morbidity-free survival of very-low-birth-weight (VLBW) infants in advanced care settings [8, 9]. In the United States, for example, Fanaroff and colleagues [10] found no significant improvement in survival without neonatal and long-term morbidity among VLBW infants between 1997 and 2002.

## 2. Anatomy and Pathogenesis of GM-IVH

The GM is located in the subependyma of the ventricular walls. It gives origin to the cerebral neuroblasts and glia, is highly cellular and gelatinous, and is richly vascularized by capillaries that are poorly supported by muscle or collagen [11]. Vascularization of the GM is prominent from 7-8 weeks of gestation and persists into the beginning of the third trimester [12, 13]. The thickness of the GM decreases after 24 weeks of gestation and almost disappears by 36–37 weeks [11]. Animal studies showed that the characteristic architecture of the subependymal matrix as the border zone between cerebral arteries and the collection zone of the deep cerebral veins makes it susceptible to focal hypoxic changes [13].

The pathogenesis of GM-IVH is intricate and multifactorial, but mostly attributed to the combined fragility of the primitive GM vasculature, fluctuations in cerebral blood flow (CBF) due to low mean arterial pressure, and impaired cerebral autoregulation in clinically unstable preterm neonates [12, 14, 15] which increases the likelihood of vascular rupture, resulting in hemorrhage that may either be restricted to the GM or extends to the adjacent lateral ventricle. Hypoxia in the GM triggers upregulation and expression of growth factors VEGF and angiopoietin-2 which induce angiogenesis. This consequently leads to formation of fragile nascent vessels that lack pericytes, display immature basal lamina low in fibronectin, and have astrocyte end-feet coverage that is deficient in glial fibrillary acidic protein [11, 16]. In addition, platelet or coagulation disorders may accentuate the hemorrhage [11]. Hemorrhagic parenchymal infarction is thought to occur when venous occlusion from a hematoma impairs perfusion in the periventricular white matter [17].

## 3. Grading of GM-IVH

**Figure 1:**
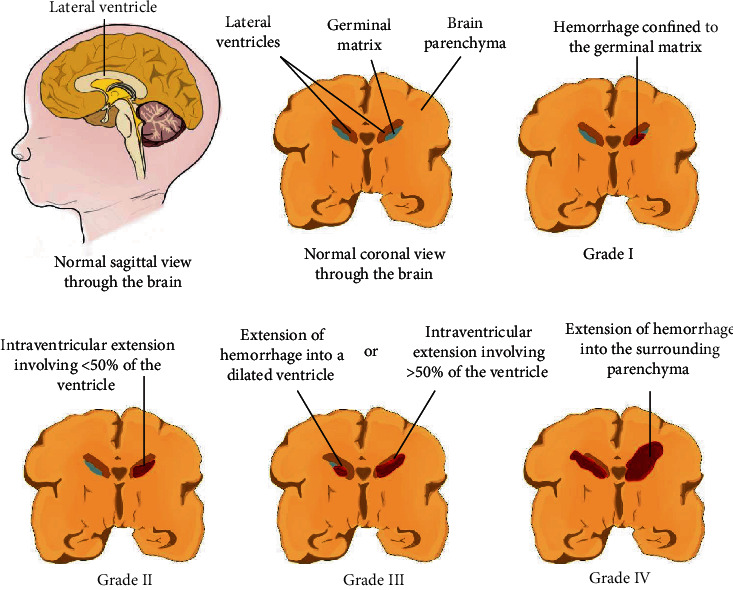
Grades of GM-IVH.

Grading systems developed by Papile et al. [18] and Volpe are the most widely accepted, although several others exist [19]. Using computed tomography scan, Papile et al. [18] developed a four-grade classification of GM-IVH based on the location and severity of hemorrhage. Grade I is defined by hemorrhage that is confined to the GM, grade II by extension of hemorrhage into lateral ventricles without ventricular dilatation, grade III when ventricular hemorrhage is present in addition to ventricular dilatation, whereas grade IV is defined by the presence of parenchymal hemorrhage [18]. A similar grading system by Volpe is based on cranial ultrasound scan (CUS). Grade I refers to hemorrhage confined to the subependymal GM, and grade II as hemorrhage within the lateral ventricle without ventricular dilation and/or hemorrhage occupying less than 50% of the ventricle. Grade III hemorrhage is defined by ventricular dilation and/or hemorrhage occupying more than 50% of the ventricle, while grade IV is ventricular hemorrhage extending into the surrounding parenchyma [20]. This is illustrated in Figure 1. Mild GM-IVH refers to grade I and II hemorrhage, while severe GM-IVH is a term used to refer to grade III and IV hemorrhage [21].

## 4. Incidence of GM-IVH

The global incidence of GM-IVH among preterm infants ranges from 14.7% to 44.7% [22–25], with considerable variation across gestational age groups, neonatal intensive care units, and countries [6, 22, 25, 26]. Hefti et al. [27] examined for GM-IVH in 345 preterm neonates autopsied from 1914 to 2015 at Boston Children's Hospital in the United States of America. The incidence of GM-IVH was 4.7% before the 1960s and increased to 50% from 1975 to 1980 following the introduction of novel positive pressure mechanical ventilation in neonatal intensive care units (NICUs), later declining by three quarters to 12.5% in 2005, probably as a result of improvements in ventilators, and the introduction of surfactant and corticosteroids. Based on age at onset, almost 40.6% of low-birth-weight (<2.5 kg) preterm neonates develop GM-IVH within the first 3 days of life, 50% by day 5, and 71.5% by day 7 [28].

## 5. Risk Factors for Development and Progression of GM-IVH

Various pre-, peri-, and postnatal factors have been implicated as independent risk factors for GM-IVH in preterm neonates. These include in vitro fertilization, absence of antenatal care, lack of maternal prenatal steroid administration, chorioamnionitis, multiple gestation, HIV exposure, fetal distress, vaginal delivery, outborn status, male gender, lower gestational age and birth weight, resuscitation at birth, delivery room intubation, anemia (low hematocrit), and blood transfusion [22, 25, 26, 28–35]. Other risk factors include clinically significant patent ductus arteriosus [36], pneumothorax [33, 37], higher fraction of inspired oxygen (FiO_2_) during the first 24 hours, early- and late-onset sepsis [31, 33], postnatal hydrocortisone administration for hypotension, inotrope use [29, 34, 38], respiratory distress syndrome requiring mechanical ventilation, hyponatremia, hyperglycemia [32], hypercapnia [36, 38], and severe metabolic acidosis [34, 39]. Studies have also indicated that preterm neonates born at lower health facilities [34] and those transferred to another hospital after birth [25, 40] are more likely to develop GM-IVH. As such, women in preterm labor should be transported to a tertiary health facility that specializes in high-risk deliveries [38]. Equally significant are genetic risk factors such as factor V Leiden (Arg506Gln), prothrombin (G20210A) gene mutations, and methylenetetrahydrofolate reductase (MTHFR 1298A>C) polymorphism [24, 41, 42]. These risk factors are summarized in Table 1.

A proportion of preterm neonates with previously diagnosed mild GM-IVH may deteriorate to severe GM-IVH. Several risk factors including maternal lower genital tract infection, lower gestational age [43], necrotizing enterocolitis (NEC), and thrombocytopenia [44] have been documented.

## 6. Clinical and Laboratory Characteristics of GM-IVH

The majority of cases of GM-IVH are clinically silent [23, 45] and only detectable by routine brain imaging. Symptomatic neonates may manifest with convulsions, bulging fontanel, recurrent apnea, unexplained pallor, respiratory distress, and temperature instability [46, 47]. Clinically identifiable seizures are reported more often among neonates with grade IV GM-IVH [48].

A significant reduction in the hematocrit may occur in the presence of a large hemorrhage [17]. Biomarkers for early prediction and detection of neuronal injury in neonates have gained clinical value in recent decades. This is because early diagnosis may provide a crucial window for implementation of neuroprotective interventions which may translate into improved outcomes. Investigators have proposed several biomarkers including S100*β*, activin A, adrenomedullin, erythropoietin, neuron-specific enolase, oxidative stress markers, glial fibrillary acidic protein, and creatine phosphokinase BB (CPK-BB). Among these metabolites, elevated S100*β* levels in the blood and urine and activin A levels in the blood are the most promising [49, 50].

## 7. Cranial Ultrasound

### 7.1. The Role of CUS

Since the late 1970s, high-resolution real-time cranial ultrasound (CUS) has been the cornerstone for diagnosis of GMH-IVH [51], with a sensitivity and specificity of 96% and 94%, respectively, for detecting intracranial hemorrhage [52]. Worldwide, CUS remains the most readily available and widely used neuroimaging modality in NICUs [53, 54]. Most importantly, CUS is portable, reliable, cost-effective, noninvasive, and radiation-free, and does not require any special preparation [53, 55, 56]. However, the findings are operator-dependent, and subtle lesions may be missed [53]. The anterior fontanelle is the most commonly used site, but an acoustic window through the posterior and mastoid fontanelles can significantly augment the findings [57, 58]. CUS can be performed at the bedside and in the incubator, within less than 5 minutes and without significant manipulation of the infant [55].

Sonographic abnormalities should be correlated on both coronal and parasagittal views, and findings on the left and right sides should be graded separately, and the location, size, and extent of the lesions are noted [59]. Interpretation of ventricular width should be done with consideration of the gestational age-specific reference ranges, as determined by Levene in 1981 [60].

### 7.2. When Should CUS Be Performed?

The timing of screening varies depending on the protocol adopted, although consensus seems to have been reached regarding the screening of all preterm neonates born before 32 weeks of gestation and/or those with VLBW [53, 58]. Nonetheless, most cases of GM-IVH occur during the first week of life [23, 28], which guides the timing of serial CUS screening. It is important to note that GM-IVH may be progressive [28], and the grade may change over time, justifying the need for CUS screening over multiple time points. In the 1980s, the initial CUS was performed during the first 3 days of life, often within 24 hours, repeated a week later among survivors, and weekly thereafter as indicated [54]. In Europe, diagnosis of GM-IVH is made by performing a bedside real-time CUS, usually on day 1, 3, 7, 14, and 28, although regular scanning may be indicated [59]. Recent Canadian guidelines recommend routine CUS for all neonates born at <32 weeks between days 4 and 7 of life or earlier depending on the clinical state of the preterm infant. Neonates born at ≥32 to <37 weeks are similarly investigated only if additional risk factors such as complicated monochorionic twin gestation, microcephaly, need for critical care, sepsis, NEC, major surgery, and/or abnormal neurological symptoms are present. Repeat imaging is performed at 4 to 6 weeks of life for all neonates born at <32 weeks and for ≥32 to <37 weeks of gestation if the first CUS result was abnormal [53]. In 2020, the American Academy of Pediatrics [58] recommended CUS for all preterm infants born at ≤30 weeks or >30 weeks of gestation with significant risk factors. The initial CUS should be performed within the first 7-10 days, with subsequent scans at 4-6 weeks of life and at term corrected age or prior to discharge. Serial CUS should be performed for infants with abnormal CUS findings, adjusted according to the clinical state.

## 8. Magnetic Resonance Imaging

Magnetic resonance imaging (MRI) is superior to ultrasound at detecting white matter abnormalities, hemorrhagic, and cystic lesions [61]. Although MRI is increasingly being utilized, it is not readily available, requires the neonate to be sedated, and may be unsuitable for unstable severely ill infants. Nonetheless, some institutions have demonstrated that MRIs may be performed without sedation of the neonate at term equivalent age [62, 63]. MRI may be performed at term corrected age for infants whose CUS reveals moderate to severe abnormalities such as grade III/IV GM-IVH, posthemorrhagic ventricular dilatation (PHVD), or grade III/IV periventricular leukomalacia (PVL), when clinical risk for white matter infarction (WMI) is increased or when parental reassurance is needed [12, 53].

## 9. Clinical Outcomes

According to Wu et al. [43], 8.2% of preterm neonates (<32 weeks) with grade II/III GM-IVH deteriorate within 7 days to grade III/IV GM-IVH. Moreover, the mortality associated with GM-IVH remains unacceptably high, even within NICUs manned by neonatologists. At least one-fifth to one-third of preterm neonates with GM-IVH die during hospitalization [24, 64], with almost 86% to 100% of deaths occurring within the first postnatal week [23, 65]. Generally, mortality increases exponentially with increasing grades [23], given that 4%, 10%, 18%, and 40% of preterm neonates with grades I-IV, respectively, die during the first hospital admission [66]. Survivors are more likely to have a prolonged duration of hospital stay, which imposes a significant financial burden to the health system [66].

Recent evidence shows that any grade of hemorrhage may be associated with abnormal neurodevelopmental outcomes, although adverse outcomes have often been linked to severe GM-IVH [2, 67–70] and lower gestational age [6, 68]. Survivors are likely to develop neurodevelopmental problems such as PHVD [71], visual and hearing impairment, severe cognitive impairment, cerebral palsy (CP), neurodevelopmental delay, and epilepsy [2, 67, 68, 70, 72, 73]. According to Christian et al. [66], 9% of preterm neonates with GM-IVH develop posthemorrhagic hydrocephalus (PHH). Among these, 1%, 4%, 25%, and 28% of patients with grades I-IV hemorrhage develop PHH, respectively. Communicating PHH accounts for most cases, thought to occur due to mechanisms such as impaired CSF reabsorption which accompanies obliteration of the arachnoid villi by microthrombi with subsequent inflammation and fibrosis [74]. Noncommunicating hydrocephalus is theorized to occur due to acute obstruction of the foramen of Monro or the aqueduct by a blood clot or due to subependymal scarring [75].

## 10. Management of GM-IVH

### 10.1. General Strategies

Management of GM-IVH is focused on addressing systemic issues of the neonate such as blood pressure and respiratory status, which might influence progression of hemorrhage. Screening for sequelae of GM-IVH should be performed, and necessary interventions are done, including management of hypotension, shock, anemia, and metabolic acidosis through judicious use of intravenous fluids and blood transfusion. Continuous EEG or amplitude-integrated EEG monitoring is indicated in the presence of seizures [17].

### 10.2. Mesenchymal Stem Cell Therapy

Animal models [76] and phase I randomized controlled trials (RCTs) involving extremely preterm infants [77] have documented the promising therapeutic potential of intraventricular transplantation of allogenic mesenchymal stem cells (MSCs) in severe GM-IVH. This novel therapy is thought to attenuate brain injury following GM-IVH and prevent the development of PHH. Current evidence is weak, and thus, more human clinical trials are needed to provide a stronger body of evidence regarding the therapeutic benefits and harms of MSCs [78]. Nevertheless, a phase 2 RCT [79] to evaluate the efficacy and safety of umbilical cord blood-derived MSCs (Pneumostem®) in 23 to <34 weeks' gestation preterm neonates with severe GM-IVH is ongoing. The primary outcomes of the study are death or shunt operation up to a postmenstrual age of 40 weeks.

## 11. Management of PHVD and PHH

Due to lack of strong evidence at the moment, there are no standardised protocols for treatment of PHVD and PHH [80], and optimal timing of interventions is still contentious [81]. Nonetheless, a low threshold for intervention has been linked to lower odds of death and poor neurodevelopmental outcomes [82]. Management of PHVD generally is aimed at preventing secondary damage due to raised intracranial pressure (ICP) and avoiding the need for a permanent shunt which may be associated with complications such as blockage and infection [71]. Several therapeutic options have been studied over decades, including conservative management, diuretic therapy, repeated cerebrospinal fluid (CSF) tapping to control excessive expansion, and drainage, irrigation, and fibrinolytic therapy (DRIFT) [72, 83].

### 11.1. Nonsurgical Strategies

#### 11.1.1. Diuretics

Available evidence has proven that medical therapy with diuretics such as furosemide and acetazolamide is inefficient, because it is associated with increased mortality and neurologic outcomes, and does not reduce the need for shunt placement [72, 84].

#### 11.1.2. Repeated Tapping of CSF

A Cochrane review of three randomized controlled trials (RCTs) and a quasi-RCT found no difference between conservative management and serial tapping of CSF via lumbar puncture or ventricular tapping as regards to reduced risk of major disability, multiple disability, death, or need for permanent shunt placement [85]. Needless to say, repeated ventricular punctures inflict a new injury to the frontal lobe with each puncture and may increase infection risk [86].

### 11.2. Surgical Strategies

#### 11.2.1. DRIFT

DRIFT involves the insertion of right frontal and left occipital catheters, with intraventricular injection of tissue plasminogen activator (e.g., urokinase) that is insufficient to produce a systemic effect [87, 88]. After 8 hours of TPA injection, irrigation with artificial CSF is commenced at a rate of 20 ml/hour, under ICP monitoring, with the goal of maintaining a pressure < 7 mmHg. The drainage fluid clears over about 72 hours, from a dark-colored thick fluid to straw-colored CSF [87]. The DRIFT approach is associated with secondary hemorrhage and does not reduce mortality neither does it alter the need for permanent shunt placement [89, 90]. Contrastingly, studies have shown a reduction in severe cognitive disability among survivors at 2 years of life [90] and at 10 years of life [91]. When performed within three weeks of IVH onset in extremely-low-birth-weight (ELBW) neonates, fibrinolytic therapy followed by external ventricular drainage may significantly reduce the need for permanent shunt surgery, without increasing the risk of secondary hemorrhage and infections [88]. Despite the shortcomings, DRIFT is cost-effective [91] and remains a suitable therapy [83].

#### 11.2.2. Shunts

Neurosurgical intervention criteria, choice, and timing of temporizing CSF diversion techniques for PHH vary across centers [81, 92]. Children with shunts from prematurity have been observed to require one or more shunt revisions and to develop slit ventricle syndrome, loculated hydrocephalus, and shunt infections more often than children with hydrocephalus due to other etiologies [93, 94].

*(1) Ventricular Reservoir*. A ventricular reservoir (VR), also known as a ventricular access device (e.g., Ommaya reservoir and McComb reservoir), is a temporizing treatment for PHH in preterm infants [86, 93, 95] that may even eliminate the need for a permanent shunt in some cases [96–98]. It involves the placement of a ventricular catheter into the right lateral ventricle that is then connected to a subcutaneous reservoir from which CSF is intermittently aspirated percutaneously to remove CSF and maintain a stable clinical state which includes normal increase of head circumference, soft fontanel, and CUS [86, 97]. As described by Kuo [86], aspiration of the reservoir is accomplished using a scalp needle of 25-gauge or smaller, with the infant in the supine position. How often and how much CSF is aspirated depends on the opening and closing pressures, respectively. VR was performed as the initial procedure in 50 (54.9%) of the 91 preterm neonates who were surgically treated for PHH at Children's Hospital Los Angeles between 1997 and 2012 [93]. As many as 57% of patients experience complications such as skin breakdown, ventricular hemorrhage, CSF infection, and leak [99]. Apnea and ventriculitis have also been documented [98]. Repeated tapping from a VR has been shown not to increase the risk of reservoir infection [95]. A prospective multicenter cohort of VLBW neonates with severe GM-IVH observed no difference in infection rates between VR and ventriculosubgaleal shunts (17% versus 14%, *p* = 0.71) [92].

*(2) Ventriculosubgaleal Shunt*. Ventriculosubgaleal shunt (VSGS) placement provides a temporary treatment of PHH in medically unstable infants and also averts the need for repeated tapping of CSF [100]. Through a small scalp incision near the anterior fontanelle, under local anesthesia and mild sedation, a ventricular catheter is carefully placed into the lateral ventricle and anchored to the dura. Blunt dissection is performed to create a pouch between the periosteum and galea, creating a subgaleal pouch where the outermost (proximal) end of the ventricular catheter is placed to allow for CSF drainage [86, 101, 102]. The procedure is described in a recent publication by Kuo [86] and can be safely accomplished in the NICU or the operating theatre [101, 103]. Collection of CSF in the subgaleal space can result in a cosmetically unappealing scalp swelling [104]. VSGS has been associated with recurrent meningitis, subgaleal adhesions, shunt obstruction requiring ventricular catheter revision or renewal, CSF leakage, and slippage of the catheter into or out of the ventricle [101, 102, 105]. It is estimated that 12% of patients with VSGS require a permanent ventriculoperitoneal shunt [101], which if needed is often placed when the CSF protein content decreases to <2 g/l, with a cell count <100 cells/*μ*l and negative CSF culture for bacteria [102].

*(3) Permanent Ventriculoperitoneal and Ventriculoatrial Shunt*. Permanent ventriculoperitoneal shunt (VPS) or ventriculoatrial shunt (VAS) placement is often performed after failure of the initial temporizing measures discussed earlier [96, 106]. Of the 21% to 36% of preterm LBW neonates with GM-IVH who subsequently develop PHH [107–109], up to 18% to 39% require permanent VPS placement [64, 66, 109]. Whitelaw and Aquilina [110] suggested VPS placement when ventricular enlargement continues at a body weight of around 2.5 kg and cerebrospinal fluid protein levels are below 1.5 g/l. On the other hand, complications associated with shunts are not uncommon, often leading to prolonged hospitalization. These include overdrainage, shunt blockage often requiring one or more shunt revisions or replacement, and infection [96, 106] predominantly caused by *Staphylococcus* species [105].

## 12. Prevention of GM-IVH

To protect the preterm brain from GM-IVH, a multifaceted approach addressing specific antenatal, delivery room, postnatal, and NICU factors should be implemented (Table 2) [111, 112]. Since GM-IVH is primarily linked to increased vascular fragility and disturbance in CBF, strategies are directed to strengthening the GM microvasculature and to stabilizing the CBF.

### 12.1. Prevent Preterm Birth

Measures that target prevention of preterm birth are the most important strategies for minimizing the occurrence of GM-IVH [21]. Preterm birth may be spontaneous or induced in situations such as eclampsia. Unless medically indicated, preterm birth can be delayed by evidence-based approaches such as antenatal progesterone supplementation from 16 to 24 weeks through 34 weeks of gestation in women with a current singleton pregnancy and previous spontaneous delivery, and those with a short cervical length (≤20 mm before 24 weeks' gestation). Other interventions such as avoidance of tobacco smoking during pregnancy, cervical cerclage for cervical incompetence, tocolytics for preterm labour, and dedicated preterm birth prevention clinics have been utilized [113, 114].

### 12.2. Prenatal Corticosteroids

The World Health Organization [115] strongly recommends prenatal corticosteroid use for all women at 24 to 34 weeks' gestation for whom preterm birth is imminent. Several studies have shown that the incidence of GM-IVH and white matter injury can be significantly reduced by the administration of a short course of prenatal corticosteroids such as betamethasone or dexamethasone [22, 31, 33, 38, 116, 117]. This protective effect may be linked to a reduction in the incidence and severity of RDS [118] and NEC [119]. Prenatal corticosteroids have also been observed to stabilize the GM vasculature through suppression of vascular endothelial growth factor and increased transforming growth factor-*β* (TGF-*β*) levels in animal studies. This results in angiogenic inhibition, trimming of neovasculature, and enhanced pericyte coverage, and consequently, a reduced propensity for hemorrhage [120].

### 12.3. Prenatal Magnesium Sulphate

Magnesium sulphate (MgSO_4_) is widely used for the prevention and management of eclampsia. A meta-analysis of 6 RCTs and 5 cohort studies conducted between 1995 and 2004 provided evidence that MgSO_4_ administered to women at high risk of preterm labor provides significant neuroprotection against moderate to severe CP, without causing adverse effects on the infants [121]. The World Health Organization, American College of Obstetricians and Gynecologists (ACOG), and the Society for Maternal-Fetal Medicine currently recommend the use of MgSO_4_ for women at risk of imminent preterm birth before 32 weeks of gestation for prevention of cerebral palsy during infancy and childhood [122, 123]. Compared to controls, MgSO_4_ has not been found to reduce the rates of GM-IVH [124].

### 12.4. Delivery at Tertiary Center and Avoidance of Interhospital Transport

Evidence from a large retrospective analysis of 67,596 VLBW preterm neonates found a correlation between interhospital transport and increased incidence and severity of GM-IVH [40], which has been linked to increased head and torso vibrations during neonatal transport [125]. A cohort study of 5,712 infants born at 24–30 weeks in the Australian and New Zealand Neonatal Network from 1995–97 found that infants transferred to another hospital after birth had 1.60 times higher odds of developing severe GM-IVH (95% CI: 1.15 to 2.21, *p* < 0.01) [22]. Therefore, when high-risk preterm delivery is anticipated, it should be conducted in a tertiary center [38, 126].

### 12.5. Delayed Cord Clumping

Delayed cord clamping (DCC) results in a higher hematocrit [127–129], superior vena cava blood flow, right ventricle output, and right ventricular stroke volume [130], higher blood pressure and admission temperature [127], less delivery room resuscitation [128], and reduced early red blood cell transfusion [131, 132]. DCC has been shown to be beneficial in preventing GM-IVH [129, 131, 132], NEC [133], and mortality [131], and can be safely implemented in singleton and monochorionic, dichorionic, and trichorionic multiple preterm gestations [134]. The optimal duration for cord clamping remains controversial. For preterm and term neonates not requiring resuscitation at birth, the American College of Obstetricians and Gynecologists, American Academy of Pediatrics, and American College of Nurse-Midwives recommend at least a 30-60-second delay to clamp the cord [135], whereas the World Health Organization strongly recommends a 60-180-second delay [136].

### 12.6. Postnatal Indomethacin or Ibuprofen

Studies performed on beagle pups [137] suggested that postnatal intravenous administration of indomethacin may confer protection against GM-IVH by stimulating basement membrane deposition in the GM microvasculature. Although early low-dose prophylactic indomethacin in VLBW preterm infants has not been independently associated with adverse neurodevelopmental function [73, 138], evidence regarding a reduction in the incidence of GM-IVH has been controversial [139–141]. One multinational RCT of extremely-low-birth-weight neonates found that early indomethacin-prophylaxis reduces the incidence of patent ductus arteriosus and severe GM-IVH [142]. Compared to the placebo group, there was no difference in adverse neurosensory outcomes at 18 months of life. In addition, a multicenter double-blind RCT showed that administration of prophylactic ibuprofen within the first 6 hours of birth was ineffective against preventing grade II to IV GM-IVH [143]. Therefore, both indomethacin and ibuprofen are not recommended for prevention of GM-IVH, but are reserved for treatment of patent ductus arteriosus.

### 12.7. Midline Head Positioning and Head Tilting

Midline (neutral) head positioning is thought to optimize cerebral venous drainage through the internal jugular veins, which are the major outflow paths for cranial blood. Head rotation to either side may result in ipsilateral occlusion or obstruction of the jugular venous drainage system [144]. Near-infrared spectroscopy (NIRS) shows that midline head position and head tilting (elevating the head of the incubator upwards by 15–30°) facilitates hydrostatic cerebral venous outflow in preterm infants [145, 146]. Moreover, Doppler ultrasonography studies showed that occlusion of the jugular venous system by changes in head position results in large alterations in blood flow velocities in the superior sagittal sinus, increased cerebral blood volume, and ICP [145, 147, 148] which may result in GM-IVH. Head positioning and tilting has been reported to have no effect on cerebral hemodynamics and oxygenation in preterm infants [149] which contrasts the findings of other studies [148]. Recent systematic reviews and meta-analyses [149, 150] reported inconclusive evidence that head positioning prevents the occurrence and extension of GM-IVH. However, a single-center study [151] found that placing <28 weeks' gestation infants in the elevated midline head position for the first 96 h of life is associated with a reduced risk of grade IV GM-IVH and mortality during hospitalization.

### 12.8. Preventing Necrotizing Enterocolitis

NEC is associated with persistently lower cerebral tissue oxygenation [152]. There is established evidence that human breast milk [153], probiotics [154], and bovine lactoferrin supplementation [155, 156] reduce the risk of NEC. The precise effects of the latter on the incidence of NEC are being studied by large multicenter RCTs such as the lactoferrin infant feeding trial (LIFT) in New Zealand, Australia [157], and Canada [158].

### 12.9. Near-Infrared Spectroscopy Monitoring of Cerebral Oxygenation

NIRS is a real-time, continuous, and noninvasive technique similar to pulse oximetry. The device uses infrared light to penetrate living tissue and estimate brain tissue oxygenation by measuring the absorption of infrared light, according to Beer-Lambert law [159, 160]. Cerebral oxygen saturation monitoring using NIRS has become a clinically useful practice because systemic arterial oxygenation does not always reflect cerebral oxygenation [161]. In a recent multicenter study of 103 neonates born at a mean gestational age of 26 weeks and birth weight < 1250 g, Chock and associates [162] found a clinically significant association between low cerebral oxygen saturation using NIRS in the first 96 hours of life and abnormal cranial ultrasonographic findings. Thus, cerebral oximetry can be used to monitor high-risk infants such that timely interventions are taken to improve cerebral oxygenation [162].

### 12.10. Ethamsylate

Ethamsylate is thought to promote platelet adhesion and increase capillary basement membrane stability through hyaluronic acid polymerization [163]. A Cochrane Database Systematic Review [164] of 1410 preterm infants from seven trials showed that infants < 35 weeks of gestation with ethamsylate are significantly less likely to develop GM-IVH compared to controls. While a significant reduction in severe GM-IVH was observed (RR 0.67, 95% CI 0.49 to 0.94), the review did not show a significant difference in neonatal mortality or neurodevelopmental outcome at two years between infants treated with ethamsylate and controls. Thus, routine use of ethamsylate for prevention of GM-IVH in preterm infants is not recommended.

### 12.11. Phenobarbitone

Earlier observations showed that phenobarbitone may dampen fluctuations in systemic blood pressure [165] and also protect the brain after hypoxia-ischemia. A 2013 Cochrane review conducted by Smit et al. [166] involved 12 controlled trials with a sample size of 982 preterm infants. In this study, the effect of phenobarbitone on the incidence of GM-IVH was controversial, with three trials reporting a significant decrease and one trial reporting an increase. Meta-analysis showed that phenobarbitone does not reduce the risk of all IVH, severe IVH, PHVD, severe neurodevelopmental impairment, or in-hospital death. Secondly, there was an increased use of mechanical ventilation in the phenobarbitone-treated group [166]. Based on this strong evidence, postnatal phenobarbitone cannot be recommended for prevention of GM-IVH.

### 12.12. Recombinant Human Erythropoietin

Early intravenous administration of high-dose recombinant human erythropoietin (rhEpo) to very preterm infants (<32 weeks) is safe and results in a significantly higher hematocrit, reticulocyte, and white blood cell counts and a lower platelet count within 7-10 days [167]. Preliminary studies by Fauchere et al. [167, 168] observed no differences between the rhEpo and placebo group with regard to the development of retinopathy of prematurity, IVH, sepsis, NEC, bronchopulmonary dysplasia, and mortality. On the other hand, studies suggest that rhEpo provides neuroprotection to ELBW and very preterm infants with IVH [169, 170].

### 12.13. Vitamin E

Vitamin E (tocopherol) is an oxidant that scavenges free radicals [163]. In 2003, Brion and colleagues [171] conducted a pooled analysis of twenty-six RCTs to evaluate the effect of Vitamin E supplementation on morbidity and mortality of preterm and LBW infants. Although vitamin E was found to reduce the risk of GM-IVH, it significantly increased the risk of sepsis in preterm infants. Among VLBW infants, the risk of severe retinopathy was reduced, whereas that of sepsis was increased, respectively. However, authors advised caution while interpreting the results, as data were heterogeneous and most included studies were conducted in the 1970s and 1980s, a time during which survival of smaller infants was low. As such, further research is required, before a recommendation can be made.

## 13. Follow-Up of Survivors of GM-IVH

Outpatient follow-up should be done to identify morbidities and provide appropriate guidance and treatment through comprehensive neurorehabilitation programs [102]. Given the increased risk of PHH, head circumference should be continually monitored [64, 72]. Children with neuropsychological deficits require special support while in school [73] with regard to writing, reading, and mathematics.

## 14. Conclusion

In recent years, considerable advances in perinatal-neonatal care have resulted in improved survival outcomes of babies born at the threshold of viability. This has been paralleled by a rising number of infants who develop complications such as GM-IVH, a multifactorial neuropathology that exclusively affects infants of ≤32 weeks' gestation or those who weigh <1500 g at birth. The GM is highly susceptible to hemorrhage due to the fragile capillary vasculature coupled with sudden fluctuations in CBF as a result of low mean arterial pressure and impaired cerebral autoregulatory mechanisms. In light of the high incidence and devastating long-term neurodevelopmental impairment associated with GM-IVH, perinatal-neonatal practitioners should optimally utilize the available evidence-based neuroprotective approaches to prevent the occurrence and extension of hemorrhage. More importantly, hospitals should adopt a protocolised schedule using serial real-time CUS to facilitate timely diagnosis of GM-IVH. Clinicians should be aware that temporary ventricular decompression can be achieved by VR and VSGS, although each has its advantages and disadvantages. There is no evidence to support the preference of one intervention technique over another for the temporary management of PHH, which highlights the need for high-quality collaborative research.

## Figures and Tables

**Table 1:** Risk factors for GM-IVH in preterm infants.

Prenatal	(i) In vitro fertilization [30, 33] (ii) No antenatal care [31, 32] (iii) Lack of prenatal corticosteroid administration [25, 29, 31, 33, 34] (iv) Chorioamnionitis [35, 36] (v) Multiple gestation [30] (vi) Low gestation age [32] (vii) Maternal HIV [28] (viii) Inherited coagulation abnormalities [24, 41, 42]
Perinatal	(i) Fetal distress [22] (ii) Vaginal delivery [25, 38] (iii) Extreme prematurity [25, 28, 36] (iv) Very low birth weight [28, 36] (v) Low 5-minute APGAR score and resuscitation at birth [25, 31, 36, 38] (vi) Intubation and mechanical ventilation [25, 31, 32, 38] (vii) Male sex [22, 26]
Postnatal	(i) Neonatal transfer after birth [22, 25, 28, 34, 38, 40] (ii) Medication (e.g., inotropes, hydrocortisone, sodium bicarbonate, normal saline boluses, and opioids) [29, 36, 38] (iii) Anemia [29] (iv) Blood transfusion [28, 32] (v) Neonatal sepsis [31, 33, 36] (vi) Patent ductus arteriosus [29, 31, 36] (vii) Respiratory distress syndrome [32, 36] (viii) Hypercapnia [36, 38] (ix) High fraction of inspired oxygen during the first 24 hours [33] (x) Pneumothorax [33, 37] (xi) Hypotension [34, 38] (xii) Hyponatremia [32] (xiii) Hyperglycemia [32] (xiv) Metabolic acidosis [34, 39]

**Table 2:** Strategies for prevention of GM-IVH in preterm neonates.

Prenatal	Perinatal	Postnatal
Prevent preterm birth Corticosteroids	Delivery at a tertiary hospital Prompt delivery upon recognition of fetal distress Delayed cord clamping	Avoid interhospital transport Elevated midline head positioning Minimize handling and stimulation Fluid therapy for hypotension Near-infrared spectroscopy monitoring of cerebral oxygenation Prevent and treat NEC and sepsis Erythropoiesis stimulation agents (e.g., erythropoietin and darbepoetin)

## Abbreviations

CBF: Cerebral blood flow
CSF: Cerebrospinal fluid
CUS: Cranial ultrasound scan
DRIFT: Drainage, irrigation, and fibrinolytic therapy
ELBW: Extremely low birth weight
GM: Germinal matrix
GM-IVH: Germinal matrix-intraventricular hemorrhage
HIV: Human immunodeficiency virus
ICP: Intracranial pressure
LBW: Low birth weight
MRI: Magnetic resonance imaging
MSC: Mesenchymal stem cells
NEC: Necrotizing enterocolitis
NICU: Neonatal intensive care unit
NIRS: Near-infrared spectroscopy
PHH: Posthemorrhagic hydrocephalus
PHVD: Posthemorrhagic ventricular dilatation
VLBW: Very low birth weight.
